# Activity of Two Antimicrobial Peptides against *Enterococcus faecalis* in a Model of Biofilm-Mediated Endodontic Infection

**DOI:** 10.3390/antibiotics10101220

**Published:** 2021-10-07

**Authors:** Giovanni Mergoni, Maddalena Manfredi, Pio Bertani, Tecla Ciociola, Stefania Conti, Laura Giovati

**Affiliations:** 1 Dentistry Center, Department of Medicine and Surgery, University of Parma, 43126 Parma, Italy; giovanni.mergoni@unipr.it (G.M.); maddalena.manfredi@unipr.it (M.M.); piobertani@piobertani.com (P.B.); 2 Laboratory of Microbiology and Virology, Department of Medicine and Surgery, University of Parma, 43126 Parma, Italy; tecla.ciociola@unipr.it (T.C.); laura.giovati@unipr.it (L.G.)

**Keywords:** antimicrobial peptides, biofilm, confocal microscopy, endodontics, *Enterococcus faecalis*, scanning electron microscopy

## Abstract

*Enterococcus faecalis* is a common cause of biofilm-associated opportunistic infections, which are often difficult to treat. The formation of *E. faecalis* biofilms on the dentinal walls of the root canal is frequently the cause of endodontic treatment failure and secondary apical periodontitis. In a preliminary work, two recognized antifungal peptides, KP and L18R, showed antibacterial activity against planktonic *E. faecalis* cells at micromolar concentrations. Moreover, L18R proved to reduce the biomass in the early stage of *E. faecalis* biofilm development on polystyrene plates, while a qualitative biofilm inhibition was demonstrated on hydroxyapatite disks by confocal laser scanning microscopy (CLSM). The aim of this study was to better characterize the effect of both peptides on *E. faecalis* biofilm. A reduction in metabolic activity after peptide treatment was detected by Alamar Blue assay, while a remarkable impairment in the architecture of *E. faecalis* biofilms on hydroxyapatite disks, along with a significant reduction in viable bacteria, was caused mostly by L18R, as assessed by CLSM and scanning electron microscopy. The lack of cytotoxicity of the investigated peptides against L929 murine fibroblasts was also determined. Obtained results suggest L18R as a promising candidate for the development of new strategies for endodontic infection control.

## 1. Introduction

Apical periodontitis (AP) is a dental pathology that involves an inflammatory lesion of the periradicular tissues caused by microbial infection of the dental pulp and biofilm formation on the dentinal walls of the root canal system [1]. Current AP treatment implies the chemo-mechanical disinfection of the root canals followed by three-dimensional obturation to prevent reinfection [2]. The most common antiseptics employed during canal irrigation are sodium hypochlorite and chlorhexidine [3]. Under clinical conditions, the efficacy of these substances is limited by the complexity of the root canal anatomy, which may limit the volume of irrigants that can reach the microorganisms [4]. Another major problem is the higher resistance of bacteria within biofilm communities to antimicrobial agents [5,6]. In particular, antimicrobial failure has been associated with reduced penetration through the biofilm matrix, biofilm-specific expression of efflux pumps, and protection against oxidative stress [5,7].

Root canal disinfection could be enhanced by placing an inter-appointment intracanal medication in order to extend the time of exposure [8,9,10,11]. The most used endodontic dressing is calcium hydroxide, Ca(OH)_2_, which inhibits the growth of many pathogens by the release of hydroxyl ions and induction of strongly alkaline conditions [12,13]. However, in the radicular environment, the activity of Ca(OH)_2_ is limited by the inherent buffer effect of dentine, and some endodontic pathogens may survive, leading to persistent infections and secondary AP [14,15]. 

Primary root canal infections are polymicrobial, dominated by anaerobic Gram-negative bacteria and composed of 10–30 species per canal [16]. In secondary, post-treatment infections, 1–5 species are detected, mostly Gram-positive facultative anaerobes [16,17,18]. In particular, *Enterococcus faecalis* is the species most frequently associated with cases of endodontic failure and persistent AP [17]. This microorganism normally inhabits the human gastrointestinal tract. However, it is a frequent cause of biofilm-associated opportunistic infections of the urinary tract and wounds, and can cause endocarditis, which are often healthcare-associated and difficult to treat due to the intrinsic resistance of *E. faecalis* to β-lactam antibiotics and the marked ability to acquire antimicrobial-resistance genes [19]. 

In the root canal environment, the inherent ability of *E. faecalis* to invade dentinal tubules, survive under unfavorable environmental conditions, such as starvation and alkaline pH, and form biofilms may contribute to its persistence after currently used treatments [20,21]. Due to these features, *E. faecalis* biofilms can be used as in vitro models to preliminarily assess the effect of novel antibiofilm agents for the development of alternative and more effective root canal disinfection strategies [22].

In recent years, there has been a growing interest in the possible use of antimicrobial peptides (AMPs) in endodontic decontamination [23]. AMPs are short, low-molecular-weight peptides of various origins, with a wide spectrum of antimicrobial activities [24,25,26,27]. The main reported mechanisms of action of AMPs involve microbial cell membrane permeabilization, but more complex interactions with diverse intracellular targets have been proposed [28].

In a preliminary work, we reported the activity of two recognized antifungal peptides, KP and L18R, against planktonic *E. faecalis* cells. Moreover, both peptides proved to reduce the biomass of *E. faecalis* biofilms on polystyrene plates, while a qualitative biofilm inhibition was demonstrated on hydroxyapatite (HA) disks by confocal laser scanning microscopy (CLSM) [29]. KP is a decapeptide derived from the sequence of the variable region of a single-chain recombinant anti-idiotypic antibody that represents the functional internal image of a wide-spectrum yeast killer toxin [30]. KP showed a remarkable activity against taxonomically unrelated pathogens, including protozoa, fungi, bacteria, and viruses [31]. L18R was synthesized on the basis of the sequence of immunoglobulin gene J (locus heavy, IGHJ2). L18R proved to display a strong fungicidal activity in vitro and to be therapeutic against *Candida albicans* experimental infection in *Galleria mellonella* [32].

The aim of this study was to better characterize the effect of KP and L18R on *E. faecalis* early-stage biofilms on polystyrene plates and mature biofilms on HA disks. Their efficacy on mature biofilms was compared with Ca(OH)_2_, a commonly used endodontic dressing. Both peptides proved to be effective against *E. faecalis* biofilms, while lacking cytotoxic activity against in vitro-cultured mammalian cells. Based on the obtained results, L18R is envisaged as a promising candidate for the development of new strategies for endodontic infection control.

## 2. Results

### 2.1. Cytotoxic Effect of The Investigated Peptides

KP and L18R were tested for their cytotoxic activity against eukaryotic cells by the MTT assay. At all the tested concentrations (up to 400 µg/mL), mean absorbance values were generally higher than those of control cells in the absence of peptides, although no statistically significant difference was observed. In Table 1, results of the cytotoxicity assay are expressed as % cell viability (control in the absence of peptides, 100% viability). 

### 2.2. Activity of KP and L18R against E. faecalis Biofilm on Polystyrene Plates 

**Figure 1:**
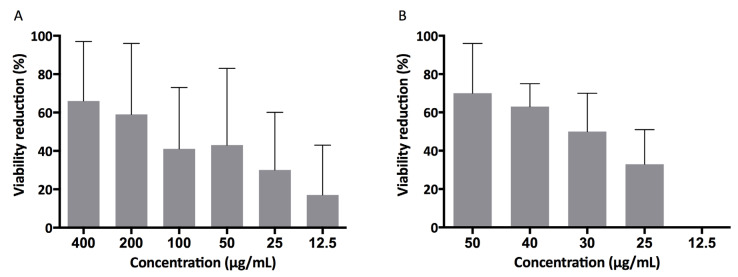
Effect of the investigated peptides against *E. faecalis* ATCC 29212 biofilm. Biofilm metabolic activity was determined by Alamar Blue assay after treatment with different concentrations of (**A**) KP and (**B**) L18R. Results are reported as percent reduction in biofilm viability as compared to untreated samples. Data are presented as mean ± SD of at least 3 independent experiments.

The capability of KP and L18R to interfere with *E. faecalis* biofilm formation on polystyrene plates was investigated by Alamar Blue assay. Both peptides proved to reduce biofilm metabolic activity in a dose-related manner (Figure 1). The half maximal effective concentration (EC_50_) value for KP was 123.3 µg/mL (confidence interval 79.41–191.6), corresponding to 1.235 × 10^−4^ M (confidence interval 0.795–1.920). L18R proved to be more active, showing an EC_50_ value of 32.77 µg/mL (confidence interval 27.72–38.73), corresponding to 1.585 × 10^−5^ M (confidence interval 1.340–1.873). 

### 2.3. Activity of KP and L18R against E. faecalis Biofilm on Hydroxyapatite Disks

**Figure 2:**
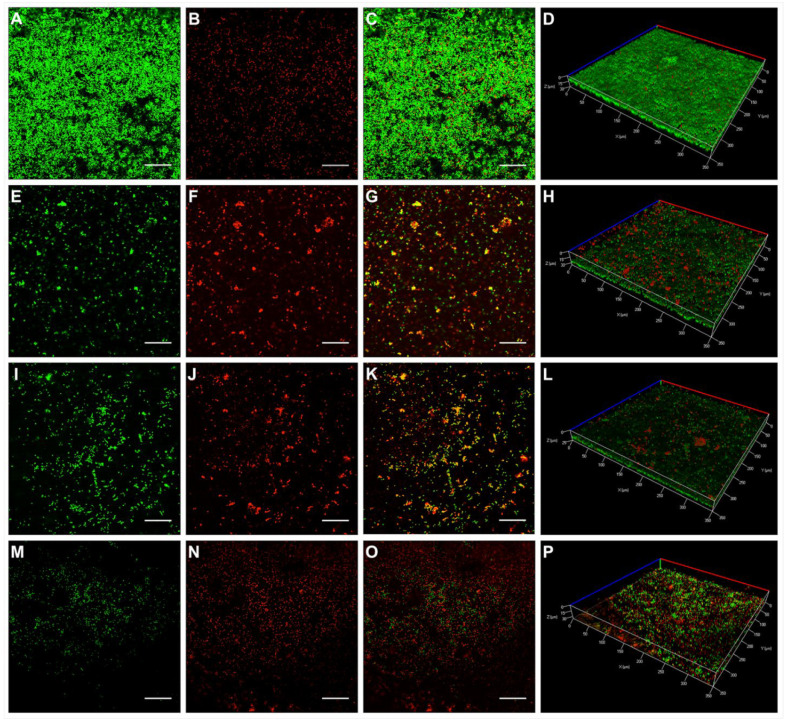
Representative CLSM images of *E. faecalis* biofilms on HA disks. Images of 48 h-old *E. faecalis* ATCC 29212 biofilm were acquired by CLSM after exposure to H_2_O (control, **A**–**C**), Ca(OH)_2_-saturated solution (**E**–**G**), KP (100 μg/mL, **I**–**K**), and L18R (50 μg/mL, **M**–**O**) for 24 h. Shown in each line is the same field (single focal plane). From left to right: SYTO-9 (green, viable cells), propidium iodide (red, dead cells), merged image of viable and dead cells. 3D reconstructions of the selected fields (full thickness, merged images) are shown in panels (**D**) (control), (**H**) (exposed to Ca(OH)_2_-saturated solution), (**L**) (exposed to KP), and (**P**) (exposed to L18R). Notably, in the L18R-treated sample, the 3D reconstruction shows a reduced thickness of the biofilm in comparison to the untreated control. Bar = 50 µm.

The activity of KP and L18R against 48 h-old *E. faecalis* biofilm grown on HA disks was assessed by CLSM and scanning electron microscopy (SEM) and compared with samples treated with a saturated Ca(OH)_2_ endodontic dressing solution. CLSM images (Figure 2A–C) and the 3D reconstruction (Figure 2D) of untreated *E. faecalis* biofilms on HA disks showed mainly viable cells organized in a homogeneous and robust biofilm layer. As compared with the untreated controls, biofilms grown on HA disks and exposed to a Ca(OH)_2_-saturated solution (Figure 2E–G), 100 μg/mL of KP (Figure 2I–K), and 50 μg/mL of L18R (Figure 2M–O) showed a consistent number of dead cells, along with fewer adhering bacteria for samples treated with Ca(OH)_2_ and L18R, as evidenced by the 3D reconstruction images (Figure 2H,L,P for Ca(OH)_2_, KP, and L18R, respectively). The reduction in biofilm thickness was particularly evident after treatment with L18R.

A quantitative analysis of the fluorescence intensities (FI), performed on the 3D CLSM reconstruction of four random fields from each disk, revealed a detaching effect of Ca(OH)_2_ and L18R treatments. In fact, the sum of total FI (viable and dead cells) was clearly reduced from 42.93 ± 4.95 for controls, to 24.18 ± 10.70 and 10.87 ± 4.33 for Ca(OH)_2_ and L18R, respectively. A different effect was observed after KP treatment, as the total FI resulted to be 76.88 ± 12. 

**Figure 3:**
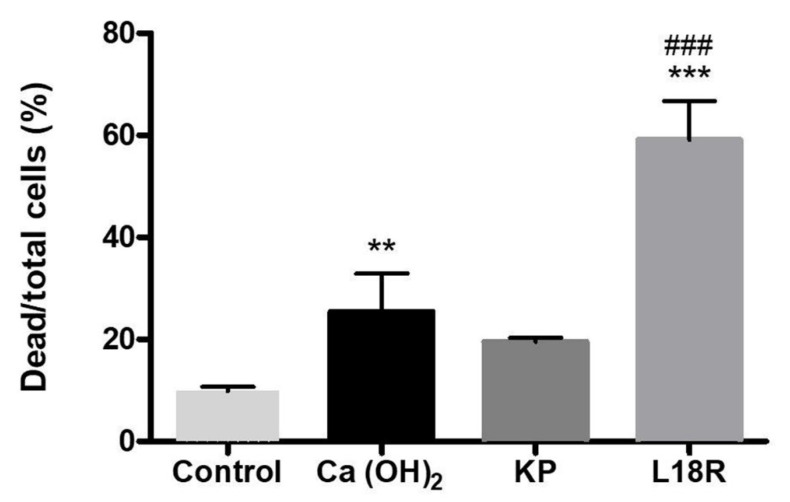
Dead cells (%) after treatment of *E. faecalis* biofilm pre-grown on HA disks. Values were obtained by 3D CLSM analysis of 48 h-old *E. faecalis* ATCC 29212 biofilms after exposure to H_2_O (control), Ca(OH)_2_-saturated solution, KP (100 μg/mL), and L18R (50 μg/mL) for 24 h. Red and green fluorescence intensity measures were carried out with Imaris 7.2 on four random fields of each disk. The results are expressed as means ± SD (**, *p* < 0.01 vs. control; ***, *p* < 0.001 vs. control; ###, *p* < 0.001 vs. Ca(OH)_2_).

The proportion of dead bacteria in the treated biofilm is shown in Figure 3. A significant increase in dead cells followed treatment with L18R and Ca(OH)_2_. Notably, in the biofilm treated with L18R, the percentage of dead cells was significantly higher than that obtained after treatment with the conventional endodontic dressing. KP treatment caused an increase, although not significant, in dead cells. 

**Figure 4:**
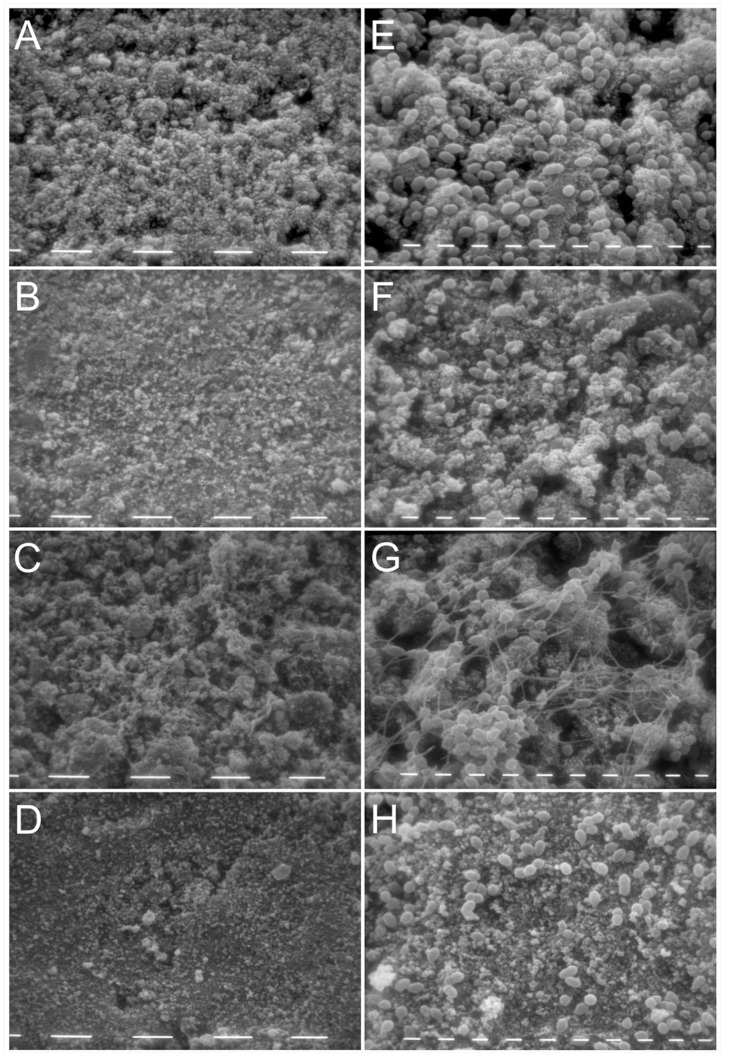
Representative scanning electron microscopy (SEM) images of *E. faecalis* biofilms on HA disks. Images of 48 h-old *E. faecalis* ATCC 29212 biofilms were acquired by SEM after exposure to H_2_O (control; **A**,**E**), Ca(OH)_2_-saturated solution (**B**,**F**), KP (100 μg/mL; **C**,**G**), and L18R (50 μg/mL; **D**,**H**) for 24 h. Magnification 1250× (**A**–**D**, bar = 10 µm) and 5000× (**E**–**H**, bar = 1 µm).

These results were confirmed by SEM images, which showed a lower number of cells in the biofilm grown on HA disks treated with Ca(OH)_2_ and L18R as compared to the untreated control sample (Figure 4). L18R treatment caused the highest detachment. In KP-treated samples, a network of fibril-like structures on adhering cells was observed. 

## 3. Discussion

In the root canal environment, the ability of *E. faecalis* to resist harsh environmental conditions and form biofilms on the inner surface of the tooth canals makes its elimination extremely difficult [21]. These features can explain the high prevalence of this bacterium in secondary and persistent endodontic infections [17]. 

In a previous published preliminary study, in order to look for new alternative and nontoxic antibacterial substances to improve root canal disinfection, the effects of the synthetic peptides KP and L18R against planktonic cells and *E. faecalis* biofilms on polystyrene plates in early-stage development were assessed [29]. The results demonstrated a good antibacterial activity of both peptides against *E. faecalis* planktonic cells at micromolar concentrations. L18R proved to be the most effective with an EC_50_ value of 3.624 × 10^−7^ M, while KP EC_50_ resulted to be 4.520 × 10^−6^ M. KP and L18R were also shown to interfere with *E. faecalis* biofilm formation by reducing biofilm mass. As for the activity against planktonic bacteria, L18R resulted to be more effective than KP in biofilm inhibition. On the basis of these promising results, new investigations were performed to better characterize the effect of both peptides on *E. faecalis* biofilms.

In the present work, the KP and L18R activities against *E. faecalis* on polystyrene plates were confirmed by the assessment of their ability to reduce biofilm metabolic activity. Notably, the comparison of the results obtained in this assay with those of the previously described experiment that evaluated the reduction in biofilm mass [29] showed a similar behavior for both peptides. In fact, taking into consideration, as an example, the concentration of 50 µg/mL, KP caused a reduction in biofilm mass of approximately 35% and in biofilm viability of approximately 43%. Similarly, L18R, at the same concentration, caused reductions in biofilm mass and viability of approximately 73% and 70%, respectively. 

KP and L18R were effective against *E. faecalis* biofilms at concentrations higher than those against planktonic cells, as commonly found with conventional antibiotics. This behavior suggests a mechanism of action, during the early phases of *E. faecalis* biofilm formation, not distinct from the killing activity against planktonic bacterial cells. On the contrary, some peptides, such as the human cathelicidin LL-37, are able to inhibit and disperse preformed bacterial biofilms at concentrations lower than or equal to concentrations effective against free-floating cells, implying an action on biofilm-specific targets rather than ubiquitous microbial structures [33]. 

From previous studies on yeasts, it has been hypothesized that the first step of KP killing activity is an interaction with cell-wall glucan-like structures [31]. For L18R, a direct penetration via an energy-independent pathway involving stable or transient destabilization and peptide folding on the lipid portion of the membrane was shown [32], indicating that the activity of this peptide may involve different mechanisms of action. Possible multi-modal mechanisms of action would render the peptides potentially advantageous in targeting different biofilm sub-populations. The mechanism of action of the investigated peptides against bacterial cells is not known, and further studies will be necessary to elucidate it.

Notably, both peptides proved to be nontoxic to murine fibroblasts at active concentrations, in agreement with previous studies that demonstrated the lack of detectable toxicity in vitro to other cell lines, erythrocytes, and peripheral blood mononuclear cells [31,32,34]. As for other AMPs, the selective antimicrobial action of KP and L18R may be explained by the cationic nature of these molecules, which promotes the interaction with the negatively charged membranes of bacteria and not with the zwitterionic membranes of mammalian cells [35]. Conversely, for other endodontic dressings of clinical use, such as Ca(OH)_2_ and cresol, a certain degree of toxicity against host cells was demonstrated [36,37].

In the preliminary study, the antibacterial effects of KP and L18R were also tested in a model of endodontic infection consisting of preformed *E. faecalis* biofilms grown on dentin-mimicking hydroxyapatite disks, in comparison with a saturated Ca(OH)_2_ endodontic dressing solution. This model, which represents a simplification of the clinical reality of the infected root canal, was used for a qualitative CLSM analysis [29].

In the present study, the same in vitro model was used for quantitative CLSM and qualitative SEM analyses. Deep alterations in the architecture and spatial distribution of the treated biofilms, with the highest detaching effect induced by L18R, were shown. In KP-treated samples, SEM images revealed a network of fibril-like structures on adhering cells, confirming the self-assembling properties previously shown by the peptide when challenged against yeast cells [31,38]. L18R treatment did not involve the formation of fibril-like structures on biofilms, according to previous observations on *C. albicans* cells [32]. It has been previously described that KP molecules easily dimerize in solution, due to the formation of disulfide bridges, and, with time, KP dimers self-assemble, giving rise to fibril-like aggregates that can be visualized by transmission electron microscopy. Moreover, KP aggregates are readily formed in the presence of soluble 1,3-β-glucans and after incubation with *C. albicans* cells exposing 1,3-β-glucans on their surface [38]. Notably, this peculiar property of KP, able to confer protection against proteases, has been associated to KP therapeutic activity in vivo against experimental fungal infections. Further studies are needed to establish which surface components on *E. faecalis* cells are able to induce KP assembly in a fibril-like network. The peculiar aggregation of KP in the presence of the targeted microorganisms could explain the data obtained by the quantitative analysis of FI in 3D reconstructions of CLSM images, i.e., the sum of total FI (viable and dead cells), which resulted to be higher for the KP-treated sample (76.88 ± 12) in comparison to the control (42.93 ± 4.95). It is conceivable that the KP fibril-like network may hinder the detachment of bacterial cells (viable and dead) during biofilm washing procedures. Likewise, the above-mentioned difference observed for KP activity against *E. faecalis* biofilms on polystyrene plates between the reduction in biofilm mass (35%) and in biofilm viability (43%), although not very high, could have the same explanation. 

The determination of the viability profile of *E. faecalis* cells allowed the detection of a significantly higher ratio of dead-to-total cells in L18R-treated samples compared to the control and to the samples treated with Ca(OH)_2_ (Figure 3). These findings suggest the potential of L18R for *E. faecalis* biofilm treatment, showing its possible benefit over the established inter-appointment medicament Ca(OH)_2_, and confirm the already reported partial resistance to the commercial endodontic dressing [39,40]. 

Further aspects, such as the spectrum of anti-biofilm activity of L18R and its activity against multispecies consortia, which may represent a greater challenge toward biofilm eradication, should be investigated. The synergistic interaction with conventional antimicrobial drugs may also be tested. Nonetheless, the obtained results indicate L18R as a promising candidate for further development as an anti-biofilm agent to be used, alone or in combination with classic endodontic dressings, as an innovative intracanal medicament to reduce endodontic failures.

## 4. Materials and Methods

### 4.1. Peptides and Bacterial Strain

KP (AKVTMTCSAS, molecular mass 998.17) was synthesized in its active dimeric form by NeoMPS (PolyPeptide Group, Strasbourg, France), while L18R (LLVLRSLGPWHPGHCLLR, molecular mass 2068.1) was synthesized at the CRIBI Biotechnology Center (University of Padua, Padua, Italy), as previously described [32,34]. Peptides were solubilized in DMSO (starting solution, 20 mg/mL) and diluted prior to use. In all experiments, controls (without peptides) contained DMSO at the proper concentration.

The reference *E. faecalis* ATCC 29212 strain was maintained in Brain Heart Infusion Agar (BHA; Sigma-Aldrich, St. Louis, MO, USA) plates. Subcultures were made two times a week.

### 4.2. Peptide Cytotoxicity Assay

The cytotoxicity of the peptides against L929 murine fibroblasts was assessed by a 3-(4,5-dimethyl-2-thiazolyl)-2,5-diphenyl-2*H*-tetrazolium bromide (MTT) assay. Cells cultured in Dulbecco’s modified Eagle’s medium (DMEM; Sigma-Aldrich, St. Louis, MO, USA) with 10% fetal bovine serum, 100 U/mL of penicillin, and 100 μg/mL of streptomycin were seeded in a 96-well microplate (100 μL/well, 4 × 10^5^ cells/mL) and incubated for 24 h at 37 °C in a 5% CO_2_ atmosphere. Cells were then treated for 24 h with the peptides at 50, 100, 200, and 400 μg/mL in DMEM with 2% FBS. Cells in medium without peptides were used as a control. The medium was discarded and 100 µL of MTT at the concentration of 0.5 mg/mL in serum-free DMEM was added in each well. After 4 h of incubation at 37 °C, 100 μL of the proper reagent (10% Triton-X 100 in acidic isopropanol 0.1 N HCl) was added to solubilize formazan crystals formed following the reduction in MTT by viable cells, and the absorbance was measured at 540 nm. Each assay was run in triplicate. Results, from two independent experiments, were expressed as the percentage of viable cells in comparison to the control. 

### 4.3. Treatment of E. faecalis Biofilm Formed on Polystyrene Surfaces

The effect of KP and L18R was investigated in early stages of the biofilm developed on polystyrene plates, as previously described [29]. Briefly, *E. faecalis* cells (7.5 × 10^6^ cells/mL, 200 μL/well) were incubated for 90 min at 37 °C; then, planktonic bacteria were removed and adherent cells were exposed to serial concentrations of peptides for 5 h at 37 °C. Cells incubated in water served as the control. After washing, the plates were further incubated at 37 °C for 48 h, and then the evaluation of biofilm metabolic activity was performed by Alamar Blue (CellTiter-Blue, Invitrogen, Carlsbad, CA, USA) assay. The plates were washed with PBS before the addition of 200 µL/well of the cell viability reagent. After 1 h incubation at 37 °C, the fluorescence was measured using a microplate reader (EnSpire^®^ Multimode Plate Reader, PerkinElmer, Waltham, MA, USA), setting excitation at 570 nm and emission at 585 nm. Each assay was run in triplicate. Four independent experiments were performed. The results were expressed as the percentage of biofilm viability reduction with reference to the untreated control (100% viability). EC_50_ values were calculated using Graph Pad Prism 4.01 software.

### 4.4. Treatment of E. faecalis Biofilm Formed on Hydroxyapatite Disks

In order to evaluate the activity of KP and L18R on mature biofilms, an in vitro model of root canal infection on hydroxyapatite (HA) disks was employed, as previously described [29]. Sterilized HA disks were placed in wells of flat-bottom 24-well plates and inoculated with 500 μL of a 7.5 × 10^6^ cells/mL bacterial suspension prepared as previously described. After 48 h of incubation at 37 °C, the medium was gently washed off and the disks were treated with 500 μL/well of a saturated solution of a Ca(OH)_2_ endodontic dressing, KP (100 μg/mL), L18R (50 μg/mL), or sterile water (control) for 24 h at 37 °C. The effect of the treatments on the biofilm preformed on HA was assessed by confocal laser scanning and scanning electron microscopy. Two independent experiments were performed.

#### 4.4.1. Confocal Laser Scanning Microscopy

For each treatment, half of the disks were examined by CLSM to determine the biofilm architecture and the viability of bacteria. After washing with PBS, bacteria on HA disks were stained using 500 μL of a live/dead kit (LIVE/DEAD FilmTracer™ Biofilm Viability Kit, Invitrogen, Paisley, UK) solution, containing two component dyes (0.3% SYTO-9, 0.3% propidium iodide), according to the manufacturer’s instructions. After 20 min, the disks were washed again and fluorescence emission was detected using a LSM 510 Meta scan head integrated with the Axiovert 200 M inverted microscope (Carl Zeiss, Jena, Germany). The excitation/emission wavelengths were 480/500 nm for the SYTO-9 live cell stain and 490/635 nm for the propidium iodide dead cell stain. The samples were observed using a 40 × NA1.3 oil immersion lens, and four random fields were scanned in each sample. A stack of 80–100 slices in 0.5 μm step sizes was captured along the Z-axis from the top to bottom of the biofilm. CLSM images were acquired and three-dimensional (3D) reconstructions were produced using the microscope manufacturer’s software (Axiovision module inside 4D release 4.5, Carl Zeiss, Jena, Germany). The ratio of red fluorescence intensity (FI) to green-and-red FI, calculated with the Imaris 9.5.0 software (Bitplane AG, Zurich, Switzerland), indicated the proportion of dead cells for treatment groups. 

#### 4.4.2. Scanning Electron Microscopy

The remaining half of HA disks were processed for SEM. Briefly, the disks were washed with PBS and dried at room temperature for 15 min. The samples were then fixed with a solution of glutaraldehyde 2.5% in 0.1 M of sodium cacodylate for 1 h at room temperature, dehydrated in graded series of ethanol (25%, 50%, 75%, 90%, 100%; 30 min between each passage), immersed in absolute acetone, and subjected to critical-point drying. The disks were mounted on aluminum stubs and covered with a 60 nm gold film using a metal sputtering device. The samples were observed using a Philips 501 microscope equipped with a Nikon Coolpix digital camera for acquisition of the images. 

### 4.5. Statistical Analysis

Statistical analysis was performed using Prism 4.01 (GraphPad software, San Diego, CA, USA). An ANOVA test followed by Tukey’s post-hoc was used for multiple comparisons. Values of *p* < 0.05 were considered significant.

## Figures and Tables

**Table 1:** In vitro cytotoxic activity of the investigated peptides against L929 cells.

Concentration (µg/mL)	Cell Viability (%) after Treatment with	
	KP	L18R
400	104 ± 17	127 ± 24
200	121 ± 15	99 ± 20
100	145 ± 34	102 ± 0
50	120 ± 39	120 ± 16

## Data Availability

The data supporting the findings of this study are available within the article.
